# The Acute Effects of Intermittent Light Exposure in the Evening on Alertness and Subsequent Sleep Architecture

**DOI:** 10.3390/ijerph15030524

**Published:** 2018-03-15

**Authors:** Minqi Yang, Ning Ma, Yingying Zhu, Ying-Chu Su, Qingwei Chen, Fan-Chi Hsiao, Yanran Ji, Chien-Ming Yang, Guofu Zhou

**Affiliations:** 1 School of Psychology, South China Normal University, Guangzhou 510631, China; minqi_yang@m.scnu.edu.cn (M.Y.); mnsas9@163.com (N.M.); yingying_zhu@126.com (Y.Z.); qingwei.chen@guohua-oet.com (Q.C.); yanran.ji@guohua-oet.com (Y.J.); 2 Department of Psychology, National Chengchi University, Taipei 11605, Taiwan; suyinchu@gmail.com (Y.-C.S.); foxlittle224@gmail.com (F.-C.H.); 3 Center for Studies of Psychological Application, Guangdong Key Laboratory of Mental Health and Cognitive Science, South China Normal University, Guangzhou 510631, China; 4 The Research Center for Mind, Brain and Learning, National Chengchi University, Taipei 11605, Taiwan; 5 National Center for International Research on Green Optoelectronics, South China Normal University, Guangzhou 510006, China; 6 Shenzhen Guohua Optoelectronics Tech. Co., Ltd., Shenzhen 518110, China

**Keywords:** acute effects, intermittent light, continuous light, dim light, alertness, sleep structure

## Abstract

Exposure to bright light is typically intermittent in our daily life. However, the acute effects of intermittent light on alertness and sleep have seldom been explored. To investigate this issue, we employed within-subject design and compared the effects of three light conditions: intermittent bright light (30-min pulse of blue-enriched bright light (~1000 lux, ~6000 K) alternating with 30-min dim normal light (~5 lux, ~3600 K) three times); continuous bright light; and continuous dim light on subjective and objective alertness and subsequent sleep structure. Each light exposure was conducted during the three hours before bedtime. Fifteen healthy volunteers (20 ± 3.4 years; seven males) were scheduled to stay in the sleep laboratory for four separated nights (one for adaptation and the others for the light exposures) with a period of at least one week between nights. The results showed that when compared with dim light, both intermittent light and continuous bright light significantly increased subjective alertness and decreased sleep efficiency (SE) and total sleep time (TST). Intermittent light significantly increased objective alertness than dim light did during the second half of the light-exposure period. Our results suggested that intermittent light was as effective as continuous bright light in their acute effects in enhancing subjective and objective alertness and in negatively impacting subsequent sleep.

## 1. Introduction

Light not only has image-forming visual effects via rods and cones, but also non-image-forming (NIF) visual effects through melanopsin-expressing intrinsically photosensitive retinal ganglion cells (ipRGCs) [1]. The ipRGCs integrate light information to the brain in two different ways: directly on their own by melanopsin and via synaptic input from rods and cones [2,3,4], and exert NIF visual functions, with 440–480 nm light being the most effective wavelengths for all NIF responses [5]. Generally, the NIF functions include circadian rhythm effects, such as resetting the timing of sleepiness-alertness and sleep-wake cycles, phase-shifting melatonin, cortisol, and core body temperature (CBT) rhythms [6], and immediate or acute effects mainly related to the inhibition of melatonin and cortisol production, enhancement of CBT and increasing alertness [3,7,8]. Thus, the influence of light on alertness and sleep includes two aspects: circadian effects and acute effects. Correspondingly, the mechanisms underlying the regulation of sleep and alertness by light include circadian clock-dependent and clock-independent pathways [2]. Specifically, the environmental light-dark cycle, via the melanopsin-based retinohypothalamic tract, entrains the endogenous timing sleep-wake system, which in turn regulates the sleep and arousal of the organism [9]. In addition, light can also act as a direct stimulant to modulate alertness and sleep by increasing brain activation [10]. 

Recently, mounting studies have examined the effects of light on subjective and objective alertness and have consistently found that exposure to light can enhance alertness [11,12,13]. For example, in the evening, exposure to monochromatic light at 460 nm for two hours induced greater subjective alertness and reduced sleepiness when compared to 550 nm exposure [14]. Additionally, in the daytime, the participants felt less sleepy and had shorter reaction times on the psychomotor vigilance task (PVT, a simple reaction time task) [13,15,16] during exposure to light (correlated color temperature: 4000 K) of 1000 lux when compared with light (4000 K) of 200 lux [17]. Furthermore, functional magnetic resonance imaging (fMRI) and positron emission tomography (PET) studies that aimed to explore the mechanisms underlying the acute effects of light on alertness confirmed that light exposure modulated alertness-related subcortical structures (hypothalamus, brainstem, thalamus) [18,19,20,21,22]. For instance, a previous study found that after light exposure (2700 K; 8000 lux), the activity in the hypothalamus decreased in proportion to the previous illumination [20]. In addition, Vandewalle and his colleagues [22] showed that white light exposure (4.16 × 10^15^ photons/cm^2^/s or >7000 lux) enhanced thalamic activity. Another study by the same team showed that monochromatic illuminations at 470 nm (3 × 10^13^ photons/cm^2^/s) typically enhanced brain responses or at least prevented the decline otherwise observed following exposure to green light (550 nm) of the same light density in the thalamus [23].

In addition to the alerting effects of light, light exposure also affects the sleep parameters. For instance, a previous study revealed that short-wavelength light is a powerful agent that immediately attenuates the sleep propensity of both homeostatic and circadian systems [18]. In addition, light exposure in the evening increased the latency to sleep onset [24] and sleep stage 2 [25,26], shortened the latency to rapid eye movement (REM) sleep [26] and the duration of REM sleep [27,28], as well as reducing sleep quality [10,28]. 

What needs to be emphasized is that the lighting pattern used in the most of these studies was continuous light, which kept the lighting parameters stable and unchanged. Only a few studies have focused on intermittent lighting patterns and this line of study has consistently shown that intermittent light elicits circadian changes that are not significantly different from continuous [29,30]. For example, phase delays of core body temperature (CBT) and melatonin rhythms in response to intermittent bright light pulses (six 15-min bright light pulses ~9500 lux were separated by 60 min of very dim light of <1 lux, and both the bright and dim light was provided by cool-white fluorescent lamps) were compared to those measured after exposure to continuous bright light of ~9500 lux. However, the acute alerting effects of intermittent lighting patterns and its effects on sleep have seldom been explored. As far as we know, only two papers have investigated this issue [31,32]. In their studies, the results showed that intermittent light (consisting of six, 15 min pulses of light of 4000 lux, with a color temperature of 3500 K) had elevated global vigor when assessed with the Visual Analogue Scale (VAS) at some time points during the daytime [31]. This might be the same case for intermittent light during the evening. 

When considering exposure to bright light, it is typically intermittent in our daily lives [33,34,35,36]. In addition, it is known that people who work in the night, such as night-shift workers, may become tired and sleepy due to the biological clock maintaining its normal “diurnal” rhythm, although the tiredness and sleepiness may be partially alleviated by bright light during the night [37]. However, whether intermittent light can be as efficient as continuous light in promoting alertness and in impacting sleep structure after light exposure remains unknown. In the current study, we aimed to explore the acute effects of intermittent light exposure during evening on alertness and the subsequent nocturnal sleep parameters (including sleep onset latency (SOL), sleep efficiency (SE), total sleep time (TST), wakefulness after sleep onset (WASO), Non-REM sleep stages (N1, N2, and N3), and REM sleep latency and duration), both of which have been frequently studied in investigations employing continuous light exposure. In order to induce greater alerting effects, we used polychromatic bright light (1000 lux) with a relatively higher color temperature (6000 K). In the present study, the first aim was to evaluate the time course of the acute alerting responses during three different patterns of light exposure (intermittent bright light, continuous bright light and dim light conditions) during the evening. Based on the findings from previous studies, we hypothesized an increase of alertness in the bright light parts of the intermittent light condition, as per the continuous bright light condition; in contrast, the alertness would be higher in the dim light parts of the intermittent light condition than that in the dim light condition. The second aim was to test whether evening exposure to intermittent bright light would affect the subsequent sleep. We predicted that evening exposure to intermittent bright light for three hours would be as effective as a three-hour pulse of continuous bright light exposure in affecting the subsequent nocturnal sleep parameters as previous studies have indicated.

## 2. Methods

### 2.1. Subjects

Fifteen healthy volunteers (mean age ± SD, 20 ± 3.4 years; range, 18–25 years; seven males) were recruited to participate in the experiment and complete the study. Eligibility for the study was assessed via a telephone and in-person interview, self-reported medical history, and a package of self-rating questionnaires. The inclusive criteria were: (1) categorized as moderately evening type, neutral type, or moderately morning type (scored 31 to 69) by the Chinese version of the Morningness-Eveningness Questionnaire (MEQ) [38]; (2) good sleeper as indicated by the score (<5) on the Chinese version of the Pittsburgh Sleep Quality Index (PSQI) [39]; (3) no indication of emotional disturbance as measured by the Chinese versions of the Beck Depression Inventory-II (BDI-II) [40], and the Chinese version of the Beck Anxiety Inventory (BAI) [41], with total scores less than 4 and 45, respectively; (4) of normal-weight (19 kg/m^2^ < BMI < 25 kg/m^2^); (5) free from medical, psychiatric, and self-reported sleep disorders; (6) no history of night work or shift work in the three years prior to study; and (7) no travelling more than two time zones in the three months prior to the study. The daily caffeine intake of all participants was kept below 300 mg, and none reported alcohol or substance abuse or tobacco use. 

Subjects were instructed to keep a regular sleep-wake schedule (bedtimes and wake-times within 1 h of self-selected schedule) during the whole week prior to each admission to the laboratory and recorded their sleep and wake times with a sleep diary. Adherence to a regular schedule was verified with an activity monitor (Actiwatch Spectrum PRO device; Philips Respironics Inc., Murrysville, PA, USA) on the non-dominant wrist. Bedtime in the laboratory was calculated from the habitual bedtime during the week before. The study was approved in September 2016 by the Ethical Committee of South China Normal University (Reference No.: 135) and carried out in accordance with the approved guidelines and regulations. All subjects confirmed their compliance through written informed consent and were paid for their participation.

### 2.2. Research Design and Procedure

**Figure 1:**
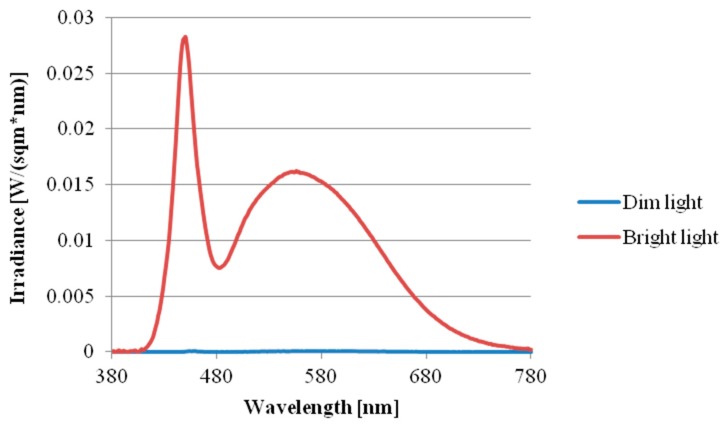
Spectral power distribution of the two light sources (bright light and dim light).

The study was a within-subject design consisting of three light conditions: the intermittent bright lighting condition (30-min pulse of bright light alternating with 30-min dim light three times, with the total time of bright light being 90 min), continuous bright light condition, and continuous dim light condition. The sequence effects of the three conditions were counterbalanced across the subjects. The duration of light exposure was three hours for all the conditions. The intensity of bright light was ~1000 lux, with dim light below 5 lux. All bright lighting throughout the study was provided by a 60 × 60 cm^2^ LED lamp panel (provided by Industrial Technology Research Institute of Taiwan, Color Temperature: 6000 K, Output Power: 40 W) top-mounted on a big box (100 × 100 × 100 cm^3^); the dim lighting was provided by a bedside lamp (3600 K, 8 W) located about 3 meters away from and behind the subjects and was the only light source in the room during the dim light condition. Light intensity was measured at eye level in the gaze direction every 30 min during the three-hour lighting session using a TES-1335 digital illuminance meter (TES Electrical Electronic Corp., Taipei, Taiwan). In addition, the spectral power distribution of the two light sources with the illuminance was measured at eye level by a calibrated spectroradiometer (JETI Specbos 1201, JETI Technische Instrumente GmbH, Jena, Germany) (see, Figure 1). The effective irradiance for each retina photoreceptors of two light resources was shown in Table 1.

Subjects were scheduled to come to the sleep laboratory for a total of four separated nights, which included an adaptation night to avoid the first night effect and three nights for the light exposure conditions. There was at least a one-week period between the three light exposure nights to avoid the residual effects of fatigue and the circadian phase delaying the effects of light.

**Figure 2:**
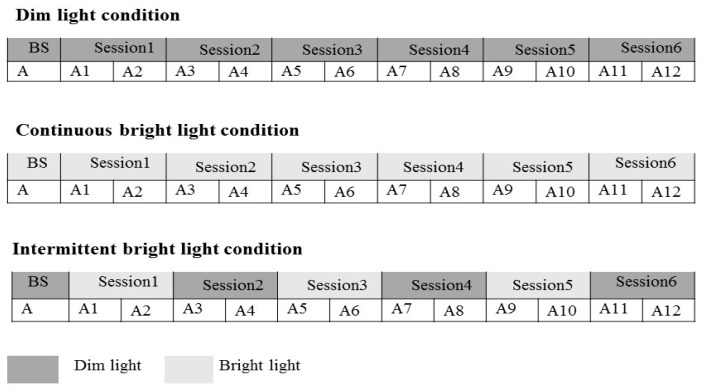
Study protocol. The timing of assessments in each laboratory session are shown in the figure. In the intermittent light condition, Session 1, Session 3, and Session 5 were bright light (1000 lux) exposure sessions, and Session 2, Session 4, and Session 6 were dim light (<5 lux) exposure sessions. In the continuous light condition, all of the six sessions were bright light (1000 lux) exposure sessions. In the dim light condition, all of the six light exposure sessions were dim light (<5 lux) exposure sessions.BS: baseline; A: assessment (PVT + KSS); A1~A12: assessment 1~assessment 12 (PVT + KSS).

The experiment was conducted during winter (between November 2016 and January 2017) to minimize the effects of outdoor light levels [43]. They stayed in a sound-attenuated room free from time cues for the three-hour duration of light exposure, while the ambient temperature was kept at 26 °C. The subjects were scheduled to come to the lab six hours before their habitual bedtime. After one hour of polysomnographic electrode placement, they remained wearing a pair of goggles for two hours to dispel the potential effects of daytime light. The baseline levels of subjective and objective alertness were assessed respectively with the Karolinska sleepiness scale (KSS) and an auditory psychomotor vigilance task (PVT) during the last 15 min of the 2-h period, followed by a 3-h light exposure period with alertness assessed every 15 min for a total of 12 times (Figure 2). They were then put to bed for sleep recording after the light exposure. 

### 2.3. Measures

#### 2.3.1. Psychomotor Vigilance Task (PVT)

Vigilance performance was assessed with computerized 10-min versions of the auditory PVT (PVT-10A) [44]. The stimulus for the PVT-10A was an auditory tone with the loudness individually tailored so that the participant had no difficulty hearing it. During the PVT-10A, subjects focused their gaze on a 5-cm black dot located on a piece of paper 100 cm away in front of the subject to make sure that each participant received the same light in each light condition. During the 10-min period, auditory tones were presented with inter-stimulus intervals of 1–9 s. Subjects were required to press the space key as soon as possible after hearing the sound. Furthermore, the lapses (reaction times greater than 500 ms) were counted as a measure of performance impairment indicative of reduced objective alertness [45,46].

#### 2.3.2. Karolinska Sleepiness Scale (KSS)

Subjective alertness was measured with a computer-administered auditory KSS, which is a widely used scale for evaluating the subjective sleepiness/alertness and has been validated against electroencephalographic (EEG) parameters [47,48,49]. KSS is a 9-point Likert scale with ratings ranging from 1 = Extremely alert to 9 = Extremely sleepy. It has been shown to be sensitive to changes in sleepiness induced by sleep loss and circadian variations, as well as the alerting effects of light [18,47,50,51,52,53].

#### 2.3.3. Sleep Recording

Nocturnal polysomnographic recording was conducted using the Embla digital system (Embla N7000 Digital PSG System, Rembrandt Embla Company, Broomfield, CO, USA) during the four sleep episodes. Scalp EEG sites C3, C4, F3, F4, O1, and O2 were paired with contralateral mastoid reference electrodes (A1, A2), based on the 10–20 system. In addition, the left and right electrooculogram (EOG), which were also referenced to A1 and A2, the chin and leg electromyogram (EMG), 2-lead electrocardiogram (EKG), nasal/oral airflow signals, chest and abdominal respiratory effort signals and oxygen saturation were also recorded. The electrode impedance was set at ≤5 KΩ for EEG and ≤10 KΩ for EMG and EKG. All signals were low-pass filtered at 35 Hz and high-pass filtered at 0.3 Hz with a sampling rate of 200 Hz (for the EEG).

Sleep stages were visually scored in 30-s epochs in the Remlogic software according to the American Academy of Sleep Medicine (AASM) Manual version 2.0.3 (AASM, Darien, IL, USA) [54]. Sleep parameters used in the current study included total time in bed (TIB), total sleep time (TST), sleep efficiency (SE, ratio of TST/TIB), percentage amount of each sleep stage (NREM sleep stage 1 (N1), NREM sleep NREM stage 2 (N2), NREM sleep stage 3 (N3), and REM sleep), which were expressed as a percentage of TST, sleep onset latency (SOL) (time from lights off until sleep onset, defined as the first of three consecutive 30-s epochs of sleep), sleep latency to REM sleep, and time of wake after sleep onset (WASO), which was expressed as a percentage of the TST.

### 2.4. Statistics Analysis

Thirteen (Testing time) by three (Light condition) repeated measures ANOVAs were conducted using SPSS Statistics version 20 (IBM, Armonk, NY, USA) to compare the subjective and objective alertness in the baseline period and during the 3-h light exposure among the three experimental conditions. Sleep parameters were also compared among the conditions with one-way repeated measures ANOVAs. The level of statistical significance was set at *p* < 0.05, with 95% confidence interval (CI). LSD correction was applied for multiple comparisons.

## 3. Results

### 3.1. Subjective Alertness

As for the KSS scores in the baseline period and during the 3-h light exposure, the results showed that both the main effect of Light conditions (*F* (2, 28) = 6.314, *p* = 0.005, *η*^2^ = 0.311) and Testing time (*F* (12, 168) = 36.636, *p* < 0.001, *η*^2^ = 0.7) reached significant levels, but the interaction effect of the two factors did not reach significant levels (*F* (24, 336) = 1.317, *p* = 0.149, *η*^2^ = 0.086). Additionally, post-hoc comparisons showed that there were no significant differences between the intermittent and continuous light conditions in the KSS scores (*p* = 0.961), but the KSS scores in the dim light condition were dramatically higher than that in the intermittent light condition (*p* = 0.013) and continuous light condition (*p* < 0.01).

**Figure 3:**
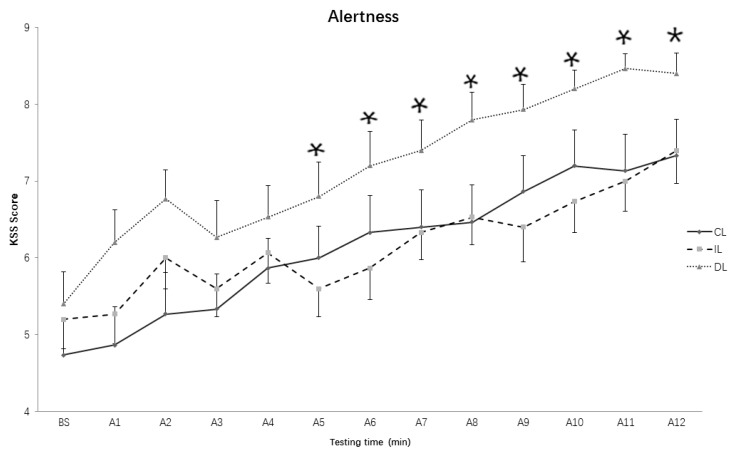
Subjective alertness over time course under different lighting conditions. CL: continuous light condition; IL: intermittent light condition; DL: dim light condition; Values: KSS raw data; Error bars: standard error; BS: baseline; A1~A12: assessment 1~assessment 12. * *p* < 0.05.

To explore the differences between the three light conditions in the subjective alertness at each assessment time, we also applied one-way repeated measures ANOVA. The results showed that there were no significant differences for all assessments between intermittent and continuous light conditions (*ps* > 0.05), but the KSS scores in the 5th–12th assessments in the intermittent light condition were significantly lower than that in the dim light condition (Figure 3). 

### 3.2. Median Reaction Time (RT) in Psychomotor Vigilance Task (PVT)

**Figure 4:**
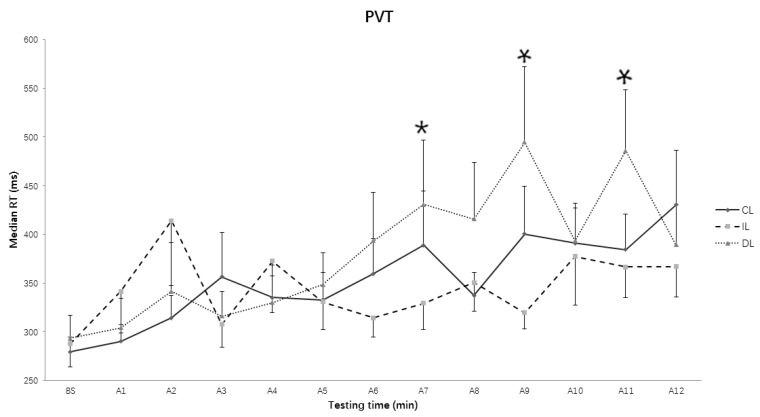
Reaction time in objective alertness over time course under different lighting conditions. CL: continuous light condition; IL: intermittent light condition; DL: dim light condition; DL: dim light condition; Values: median RT; Error bars: standard error; BS: baseline; A1~A12: assessment 1~assessment 12. * *p* < 0.05.

The two-way repeated ANOVA results showed that the main effect of Light condition (*F* (2, 28) = 2.25, *p* = 0.124, *η*^2^ = 0.138) did not reach significant levels, but the main effects of Testing time (*F* (12, 168) = 3.855, *p* = 0.003, *η*^2^ = 0.216) and the interaction of Light condition and Testing time did reach significant levels (*F* (24, 336) = 2.221, *p* = 0.001, *η*^2^ = 0.137). Simple test analysis showed that the RT in the intermittent light condition was significantly shorter than that in the dim light condition in the 7th assessment (*p* = 0.035), 9th assessment (*p* = 0.030), 11th assessment (*p* = 0.013) (Figure 4), but there was only a trend that the median RT in the continuous light condition was longer than that in the intermittent condition (*p* = 0.051) on the 9th assessment, and shorter than that in the dim light condition on the 11th assessment (*p* = 0.054). Additionally, there was a significant decrease in the median RT from the 11th to the 12th assessment (*p* = 0.037) in the dim light condition. 

### 3.3. Lapses of RT in PVT

**Figure 5:**
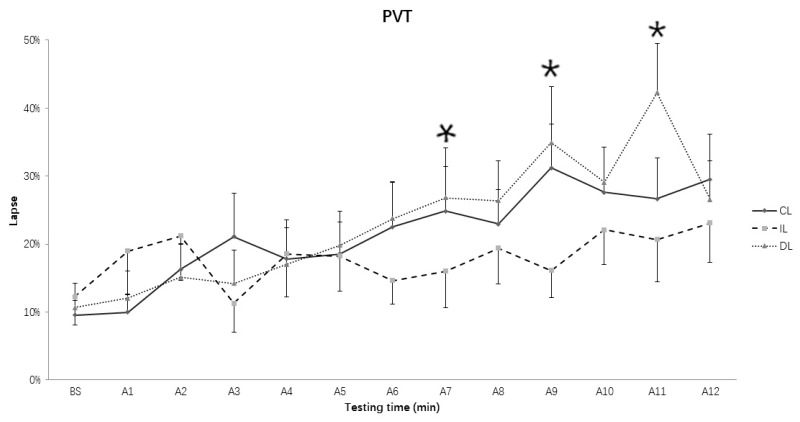
The lapse of Psychomotor Vigilance Task (PVT) over time course under different lighting conditions. CL: continuous light condition; IL: intermittent light condition; DL: dim light condition; Values: the percent of lapse number; Error bars: standard error; BS: baseline; A1~A12: assessment 1~assessment 12. * *p* < 0.05.

The two-way repeated ANOVA results on the number of lapses showed that the main effect of Testing time (*F* (12, 168) = 7.452, *p* < 0.001, *η*^2^ = 0.347) and the interaction of Light condition and Testing time (*F* (24, 336) = 2.055, *p* = 0.003, *η*^2^ = 0.128) did reach significant levels, but not the main effect of Light condition (*F* (2, 28) = 2.367, *p* = 0.112, *η*^2^ = 0.145). Simple test analysis showed that the number of lapses in the intermittent light condition was significantly less than that in the dim light condition in the in the 7th, 9th, and 11th (*ps* = 0.027, 0.033, and 0.001, respectively) (Figure 5), and less than that in the continuous bright light condition in the 7th (*p* = 0.015) and 9th (*p* = 0.002) assessments. In addition, the number of lapses in the continuous bright light condition was significantly less than that in the dim light condition in 11th assessment (*p* = 0.041). Consistent with the median RT, the lapse in the 12th assessment showed a significant decrease when compared to the lapse in the 11th assessment in the dim light condition (*p* = 0.021). 

### 3.4. Sleep Structure

A one-way ANOVA with the factor “Light condition” indicated that the intermittent and continuous light exposures had negative effects on some sleep parameters such as SE and TST, when compared with dim light exposure (Table 2).

### 3.5. Total Sleep Time (TST)

There were significant differences between the three conditions in the TST (*F* (2, 28) = 3.473, *p* = 0.045, *η*^2^ = 0.199). Post-hoc analysis showed that the TST following the dim light condition was significantly longer than that following the intermittent condition (*p* = 0.031), but there was only a trend that the TST in the dim light condition was longer than that in the continuous light condition (*p* = 0.07), and the TST in the continuous condition was not significantly different from that in the intermittent light condition (*p* = 0.571).

### 3.6. Sleep Efficiency (SE)

The difference in the SE between the three light conditions reached significant level (*F* (2, 28) = 4.5, *p* = 0.02, *η*^2^ = 0.243). Post-hoc comparisons showed that the SE in dim lighting condition was significantly higher than the intermittent lighting (*p* = 0.02) and continuous bright conditions (*p* = 0.018), but there was no significant difference between the intermittent bright light and continuous bright light condition (*p* = 0.297). 

### 3.7. Time in Bed (TIB) and Ratio of Different Sleep Stages

There were no significant differences among the three lighting conditions in the total time in bed (TIB) (*F* (2, 28) = 0.619, *p* = 0.546, *η*^2^ = 0.042), and the ratio of N1 (*F* (2, 28) = 0.131, *p* = 0.878, *η*^2^ = 0.009), N2 (*F* (2, 28) = 0.583, *p* = 0.565, *η*^2^ = 0.040), N3 (*F* (2, 28) = 0.156, *p* = 0.856, *η*^2^ = 0.011), and REM sleep in total sleep time (*F* (2, 28) = 1.099, *p* = 0.347, *η*^2^ = 0.073). 

### 3.8. Rapid Eye Movement (REM) Sleep Latency from Sleep Onset

The three lighting conditions did not produce differences for the REM latency from Sleep Onset (*F* (2, 28) = 2.332, *p* = 0.116, *η*^2^ = 0.143).

### 3.9. Sleep Onset Latency (SOL)

There was a tendency that the three lighting conditions elicited differences for SOL (*F* (2, 28) = 3.275, *p* = 0.074, *η*^2^ = 0.190). Post-hoc comparisons showed that the SOL in the dim lighting condition was significantly shorter than that in the continuous lighting condition (*p* = 0.002), but not different from that in the intermittent lighting condition (*p* = 0.062), and there was no significant difference in SOL between the intermittent light and continuous bright light conditions (*p* = 0.781).

### 3.10. Wake after Sleep Onset (WASO)

There was also a non-significant trend where the three lighting conditions induced differences for the ratio of WASO in TST (*F* (2, 28) = 2.813, *p* = 0.077, *η*^2^ = 0.167). Further analysis revealed that the WASO in the intermittent lighting condition was significant larger than that in the dim light conditions (*p* = 0.04), but the WASOs in the continuous bright condition were not different from that in the intermittent (*p* = 0.177) and dim light conditions (*p* = 0.432). 

## 4. Discussion

The current study compared the effects of intermittent bright light, continuous bright light, and continuous dim light on subjective and objective alertness during the exposure and the sleep structure after exposure. Our data indicated that, as expected, the subjective alertness levels decreased over time for all conditions and were higher during the two conditions with bright light exposure than the dim light condition. The objective measure of alertness, however, was significantly higher in the intermittent light condition than in dim light condition, even significantly higher than in the continuous light condition in one assessment, during the second part of the light exposure. Besides that, when compared with dim light exposure, both intermittent light and continuous bright light exposures decreased TST and SE but showed minimum effects on the alteration of sleep architecture. In addition, there was no significant difference in the sleep structure between intermittent and continuous bright light conditions.

It was surprising that the effects of the dim light (<5 lux) pulses in the intermittent light condition on subjective alertness were not significantly different from the effects of continuous bright light, with the subjective alertness being significantly higher in the intermittent and continuous light conditions than in the dim light condition. This result indicated that the alerting effects could outlast light exposure, which was consistent with the study by Smolder and colleagues [17], which suggested that bright light (1000 lux at eye level) exposure had an immediate and persistent effect on subjective alertness. These results were supported by previous findings on the pattern of activation of ipRGCs and SCN neurons, both of which are involved in the alerting effects of light. For instance, Dacey and colleagues [55] showed that light pulses of a few seconds could significantly induce a sustained response that outlasted the light stimulus, and which declined slowly in ipRGCs. Furthermore, in rodents, the irradiance detection response was not constant in the firing rate in SCN neurons, with the rate typically increasing during light exposure but showing a substantial and prolonged (i.e., several minutes) undershoot when the light was switched off [20]. Findings from fMRI studies further demonstrated the light-induced modulation of brain responses induced by and outlasted light exposure. For example, the alerting effect of 30-min bright light (6500 lux) exposure could be maintained for up to 10 min after lighting termination [22]. 

As for the objective alertness across time, our data indicated that the subjects in the intermittent light condition performed significantly better in the PVT in the assessments following the switching between dim light and bright light during the second half light exposure period than that in dim light, even in continuous light conditions. In addition to the aftereffects of light on alertness, it was likely that the alerting effects of bright light could also be attenuated with continuous exposure and could be facilitated after a period of the removal of bright light. The current findings were consistent with previous studies that demonstrated that light history could lead to a sensitivity of the photoreceptive system. That is, higher sensitivity to the initial few minutes of bright-light exposure may result from prior exposure to very low light levels [35,56,57,58]. For instance, melatonin suppression induced by 200 lux light in an approximately 0.5 lux prior light history condition was found to be greater than that in the approximately 200 lux background condition [6].

In the current study, the performance in the PVT was better in the bright light (including continuous and intermittent light) conditions in the later part of light exposure when compared with dim light conditions. The result was consistent with a previous study showing that the effects of bright light on subjective sleepiness were stronger under mental fatigue [57]. In addition, Rahman and his colleagues [14] suggested that blue light improved alertness by reducing the effects of homeostatic sleep drive both during the day and night, but enhanced nighttime alertness by an additional increase in the circadian drive for alertness. In our study, since the latter part of the 3-h light exposure was relatively closer to bedtime, which was accompanied with larger homeostatic and circadian drives, the increased level of alertness induced by light could have resulted from the increase of both the effects of homeostatic and circadian sleep drive. The reason why there was no significant difference between the intermittent and dim light conditions in both the subjective and objective measures of alertness in the earlier part of light exposure may be due to the adjustments of participants to the lighting environment in the beginning period of light exposure. Alternatively, it is possible that the first part of the light exposure was in the wake maintenance zone [59] when the subjects were still in an alert state. Therefore, there was a ceiling effect that limited the possibility of revealing a treatment effect.

Although both the subjective and objective measures of alertness were increased by bright light, it seemed that the subjective alertness was more sensitive to the light. This was consistent with a study that showed that the effects of illuminance on the subjective measures were not dependent on the duration of exposure, but the enhancement of objective alertness induced by light were most pronounced towards the end of the one-hour exposure period [17]. That is, the objective and subjective alertness were not synchronized with each other, with the objective alertness enhancement induced by light lagged behind the improvement in the subjective alertness. 

It is to be noted that the effects of intermittent light on the alertness revealed in the current study indicate that the intermittent light may be a better choice for people who are sleepy and need promoting alertness, such as night-shift workers who are typically sleepy during night-shift work [60] and people who complain of drowsiness in the daytime. Firstly, because the intermittent light is of high efficiency for enhancing alertness and decreasing sleepiness, which was demonstrated in the present study. In addition, intermittent light could enhance the adherence to the light therapy; that is, it did not need the individual sit in front of bright light continuously for a long time.

As for the sleep parameters, our results showed that bright light exposure (including intermittent and continuous light) in the evening significantly disrupted sleep by decreasing SE and TST, which was consistent with previous studies showing that white LED exposure during the night could cause poor sleep quality and quantity [61,62]. In the current study, the negative effects of three hours of continuous bright and intermittent light exposures on sleep were in parallel to the effects on the objective and subjective alertness. Therefore, the significantly negative influences of intermittent light exposure on subsequent sleep could be due to the alerting effect of light that persisted into the sleep episode [27]. In addition, considering the negative effect on sleep, that is, decreasing the SE and TST, revealed in the present study, we also suggest that just like the suggestions for continuous light exposure before bedtime, the intermittent light exposure prior to sleep in daily life also should be minimized if possible, especially for people who have complaint of low sleep efficiency, such as insomniacs [63]. 

## 5. Limitations

In light of the significance of our findings as mentioned above, the results should be interpreted with caution due to the following limitations. First, the sample size of our study was relatively small, which might limit the statistical power to detect the possible effects of the experimental manipulation. Future studies are needed to validate the findings. Second, thirteen 10-min PVTs were conducted over the period of three hours and 15 min before the subjects went to bed for sleep. Such an intensive workload might have resulted in greater energy expenditure and led to higher homeostatic sleep pressure as reflected by the short sleep onset latencies in all three conditions. This heightened sleep drive might have hampered the capacity to detect differences in the effects on sleep parameters among the three conditions. In addition, circadian phase markers and other indicators of the central nervous system, both of which could help reveal the mechanisms underlying nocturnal sleep comprehensively and systematically, were not measured. Furthermore, in the current study, the bright light administered was designed to be sufficient to generate an effect. The intensity was therefore higher than the lighting in daily life conditions. It is not certain whether the findings could be generated by lighting of lower intensity, shorter duration, or different color temperatures. Furthermore, even though the subjects wore a pair of goggles for two hours to dispel the effects of daytime light in the experimental day before the evening light exposure in the current study, we did not control the light history of previous weeks or months. Finally, the present study only tested the effects of light during the following sleep period. Future studies could explore the possible impact of light on the daytime functions the next day and on the next night’s sleep. 

## 6. Conclusions

As hypothesized, our study showed that intermittent light was equally as effective as continuous light in enhancing subjective alertness, and even more effective in increasing objective alertness during the latter part of light exposure when the subjects were relatively sleepy. In addition, intermittent light exposure was as effective as continuous bright light in disrupting the sleep structure after light exposure, with decreases in SE and TST. These effects on sleep are probably continuations of the alerting effects of bright light prior to sleep. These findings indicate that the use of intermittent light may be a better choice for people who are sleepy and need promoting alertness, such as night-shift workers [62] and people who complain of drowsiness in the daytime, because of the effective alerting effects and individuals’ relatively higher adherence of intermittent light exposure. It also suggests that intermittent light exposure prior to sleep in daily life might have negative impacts on subsequent sleep and should be minimized before bedtime if possible, especially for people who have complaint of low sleep efficiency, such as insomniacs [63].

## Figures and Tables

**Table 1:** Spectrally weighted α-opic illuminance levels for each lighting condition.

Sensitivity	λmax (nm)	α-Opic Lux Value (~5 Lux)	α-Opic Lux Value (~1000 Lux)
Melanopsin	480.0	2.27	870.70
S-cone	419.0	1.46	911.50
M-cone	530.8	3.98	939.15
L-cone	558.4	4.45	920.03
Rods	496.3	2.92	903.13

Note: the five α-opic irradiances were determined by using the calculation toolbox developed by Lucas et al., (2014) [42].

**Table 2:** Sleep parameters subsequent to the three lighting exposure conditions.

Stages	IL	CL	DL	*F*	*p*	*η*^2^
TIB (min)	393.9 ± 17.0	392.3 ± 16.2	404.1 ± 13.8	0.619	0.546	0.042
TST (min)	363 ± 15.9	368.4 ± 15.0	387.5 ± 13.3	4.473	0.045	0.199
SOL (min)	13.1 ± 3.7	9.6 ± 1.8	4.4 ± 0.7	3.782	0.063	0.213
RL (min)	69.1 ± 7.1	81.4 ± 7.6	66.7 ± 3.2	2.332	0.116	0.143
SE (%)	92.3 ± 1.4	94.0 ± 0.8	95.9 ± 0.4	3.894	0.049	0.218
N1 (%)	4.2 ± 0.5	4.4 ± 0.9	4.1 ± 0.6	0.131	0.878	0.009
N2 (%)	55.6 ± 1.5	56.3 ± 1.3	54.9 ± 1.8	0.583	0.565	0.040
N3 (%)	16.0 ± 1.6	16.7 ± 1.7	16.6 ± 1.2	0.156	0.865	0.011
REM (%)	24.1 ± 1.3	22.6 ± 1.2	24.5 ± 1.2	1.099	0.347	0.073
WASO (%)	5.2 ± 1.1	2.7 ± 0.5	2.8 ± 0.4	4.800	0.077	0.286

CL: continuous light condition; IL: intermittent light condition; DL: dim light condition; TIB, time in bed; TST, total sleep time; SOL, sleep latency to sleep onset; RL, REM sleep latency (after sleep onset); SE, sleep efficiency (TST/TIB × 100); N1, NREM sleep stage 1; N2, NREM sleep stage 2; N3, NREM sleep stage 3; REM, REM sleep; WASO (%), the time of wakefulness after sleep onset in the TST. Values are depicted as mean ± 1 standard error of the mean (*N* = 15).
